# Knowledge, attitude, and use of protective measures against COVID-19 among nurses: a questionnaire-based multicenter cross-sectional study

**DOI:** 10.1186/s12912-021-00689-x

**Published:** 2021-09-07

**Authors:** Ramzi Shawahna

**Affiliations:** 1 Department of Physiology, Pharmacology and Toxicology, Faculty of Medicine and Health Sciences, An-Najah National University, Building: 19, Office: 1340, P.O. Box 7, Nablus, Palestine; 2 An-Najah BioSciences Unit, Centre for Poisons Control, Chemical and Biological Analyses, An-Najah National University, Nablus, Palestine

**Keywords:** Awareness, Knowledge, Attitude, Protection, COVID-19, Nurses

## Abstract

**Background:**

During this pandemic, nurses have always been on the frontline and are probably the first healthcare providers to interact with patients presenting with symptoms of COVID-19. The main aim of this multicenter study was to assess knowledge, attitude, and use of protective measures against COVID-19 among nurses across the Occupied Palestinian Territory (oPt) during the ongoing pandemic.

**Methods:**

This was a questionnaire-based multicenter cross-sectional study that was conducted in the period between October 2020 to December 2020. The study tool tested knowledge (8-item), attitude (2-item), and use of protective measures against COVID-19 (3-item) among nurses. Associations between nurses’ characteristics and their knowledge, attitude, and use of protective measures were investigated using Student’s t-test, Analysis of Variance, and Pearson’s correlations. To control potentially confounding variables, predictors of higher knowledge, attitude, and use of protective measures were identified using multiple regression analyses.

**Results:**

The study tool was complete by 455 nurses. The mean of knowledge, attitude, and use of protective measures scores were 75.7% (SD:12.4%), 75.1% (SD: 17.7%), and 91.6% (SD: 18.2%), respectively. Multiple linear regression models showed that high knowledge was predicted by being female (*p*-value = 0.004) and self-rating social status as high (*p*-value = 0.005). Higher attitude was predicted by being female (*p*-value = 0.005), self-rating academic achievements as high (*p*-value = 0.007), and having contracted COVID-19 (*p*-value = 0.001). Higher use of protective measures was predicted by self-rating academic achievements as high (*p*-value = 0.010).

**Conclusion:**

Findings of this study suggested that nurses in the oPt had high knowledge, relatively optimistic attitude, and appropriately used protective measures against COVID-19 during the ongoing pandemic. Knowledge, attitude, and use of protective measures among nurses should continuously be updated as information unfold during the ongoing pandemic. More efforts are still needed to ensure protection of healthcare providers including nurses from contracting COVID-19.

**Supplementary Information:**

The online version contains supplementary material available at 10.1186/s12912-021-00689-x.

## Introduction

The severe acute respiratory syndrome coronavirus 2 (SARS-CoV-2) or novel coronavirus 2019 (2019-nCoV) that causes coronavirus disease 2019 (COVID-19) was first reported in Wuhan, China in December 2019 [1]. Later, the disease spread to almost every country in the world. On March 11, 2020, the director-general of the World Health Organization (WHO) has declared the outbreak of COVID-19 as a global pandemic [2, 3]. As of December 14, 2020, there were 71,051,805 confirmed cases that included 1,608,648 loss of lives reported to the WHO on a global level [4]. On March 5, 2020, the first case of COVID-19 was diagnosed in the Occupied Palestinian Territory (oPt) [5]. The authorities in the oPt immediately responded by declaring a state of emergency and containment measures that included a lockdown, restricting movement, and closure of all non-essential establishments. As of December 14, 2020, there were 126,205 confirmed cases including 1107 deaths in the oPt according to the statistics of the Palestinian Ministry of Health [6]. With regard to the clinical features, COVID-19 does not seem very significantly different from severe acute respiratory syndrome (SARS) caused by (SARS-CoV). Studies have demonstrated that the fatality rate of COVID-19 was significantly lower than that of SARS (2.3% vs 9.5%) and that of Middle East respiratory syndrome (MERS) (2.3% vs 34.4%) [7]. On the other hand, the reproductive number (R_0_) and infection kinetics showed that SARS-CoV-2 was more contagious than SARS-CoV (2.0–2.5 vs 1.7–1.9) and MERS-CoV (2.0–2.5 vs < 1) [7, 8]. The main transmission routes of COVID-19 are airborne droplets, direct contact with an infected individual, direct contact with surfaces, and/or objects contaminated by body fluids of an infected person [9].

Because healthcare providers are in direct and prolonged contact with infected patients, they are at an increasing risk of contracting the disease. Infections, need for hospitalization, and/or isolation of healthcare providers have led to depleting the healthcare workforce in different countries around the world [10, 11]. During this pandemic, many healthcare facilities around the world faced shortage of healthcare providers, beds, personal protective equipment, and other medical supplies. As a result, healthcare providers had to face work overload and significant levels of burnout [12–14]. In all healthcare systems around the world, nurses are the providers of the largest volume of healthcare services to patients. During the pandemic, nurses in all healthcare system were on the front-line during the fight against COVID-19 [15]. Because nurses are the first healthcare providers to interact with the patients presenting with symptoms, they are at a higher risk for contracting the disease [15–17]. Studies from different regions of the world have reported healthcare centers being hit by COVID-19 and many healthcare providers including nurses testing positive [18, 19]. Therefore, there has been many calls to support nurses and protect them from contracting the disease [10, 11, 15, 20–22].

Recent studies assessed knowledge, attitude, and use of protective measures against COVID-19 among healthcare providers including nurses in different healthcare systems around the world [23–32]. A recent study in Jordan showed that the precautionary behavior among medical doctors during the ongoing COVID-19 pandemic was not optimal [33]. Another study showed that Jordanian nurses perceived their role as constructive during the ongoing pandemic [34]. The nurses supported and advocated for the patients and their caregivers despite the increasing workload during the ongoing pandemic.

It has been argued that adequate knowledge supported by positive attitude might lead to appropriate use of protective measures at work. This might subsequently decrease the risk of contracting the disease [35]. Probably, adherence of nurses to using protective measures against COVID-19 might be affected by their knowledge and attitude toward the disease. Therefore, assessing knowledge, attitude, and use of protective measures against COVID-19 among nurses could be of crucial importance. Additionally, understanding factors the affect knowledge, attitude, and use of protective measures against COVID-19 might be important for designing future interventions to protect nurses from contracting COVID-19 and other contagious diseases.

Little is known on knowledge, attitude, and use of protective measures against COVID-19 among nurses in the oPt. The main aim of this multicenter study was to assess knowledge, attitude, and use of protective measures against COVID-19 among nurses during this ongoing pandemic. Another objective was to identify the factors that could be associated with high knowledge, optimistic attitude, and adequate use of protective measures. As protecting healthcare providers, notably, nurses has become a priority, this study was conducted in the context of understanding the current behavior.

## Methods

### Study design and setting

**Fig. 1:**
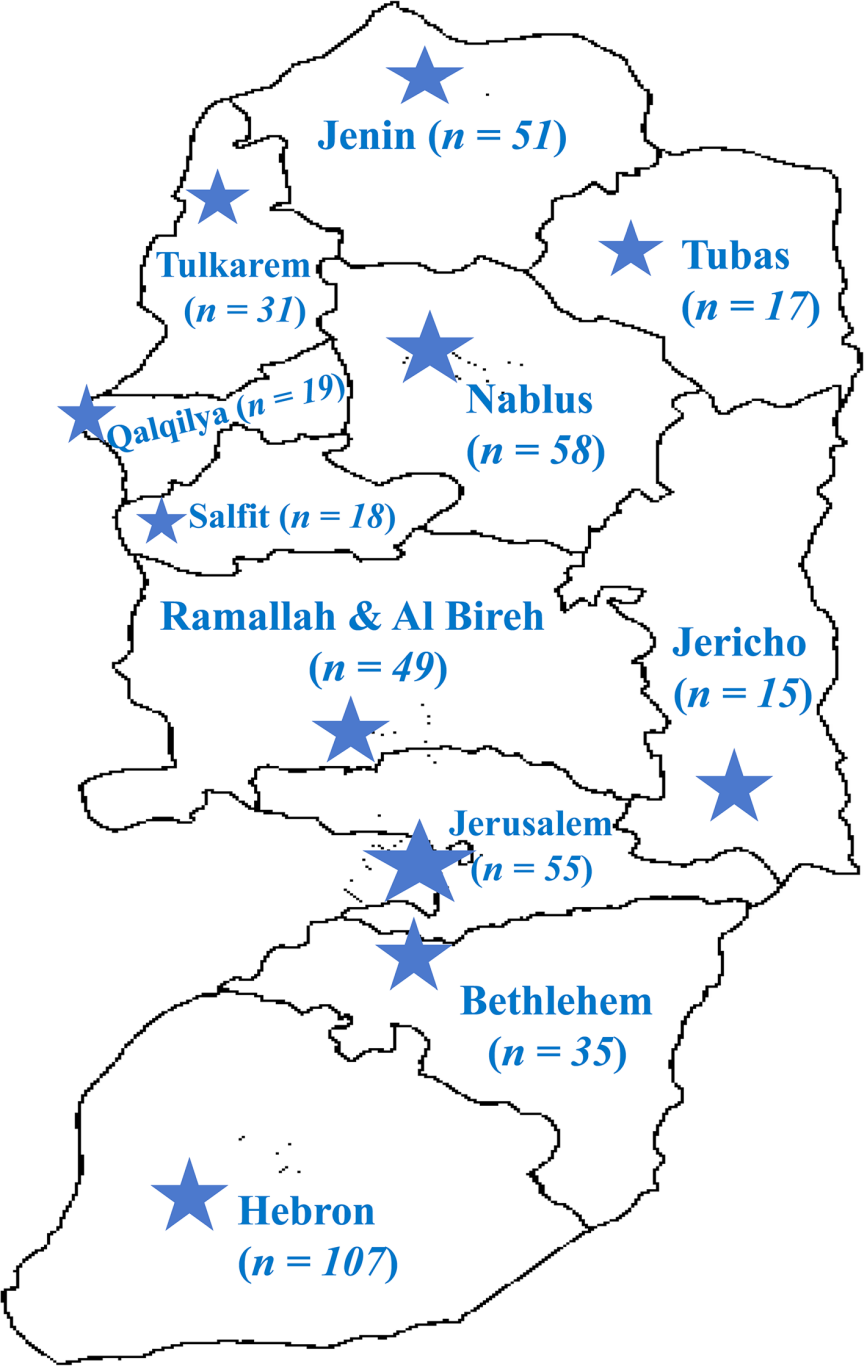
Healthcare centers/hospitals from where the nurses were recruited (the map was adopted and modified from Wikimedia Commons that can be accessed from: http://commons.wikimedia.org/wiki)

The oPt has been affected by the ongoing COVID-19 pandemic since March 5, 2020. Later, cases were reported in all regions and governorates. This study was a cross-sectional survey that was conducted among nurses in the oPt from October 2020 to December 2020 utilizing a paper-based questionnaire. The study involved nurses from multiple healthcare centers/hospitals from all governorates in the oPt (Fig. 1). The study is reported in adherence to the guidelines for reporting cross-sectional studies in which a questionnaire was used as the study tool (Supplementary Table S1) [36–38].

### Study participants and sampling

The target population in the present study was nurses who practice their jobs in various healthcare institutions in the oPt during the ongoing pandemic. A sample size calculator that is freely accessible online at (www.raosoft.com) was used to compute the sample size to be recruited in this study. Assuming a maximal population of 20,000 nurses practicing in the oPt during the ongoing pandemic, the sample size was computed at a 95% confidence interval (CI) with a margin of error of ≤5%. The sample size to be included in this study was estimated as 385 nurses. Quota sampling was used to recruit the study participants for this study. Quotas were proportionate to the population of each governorate. The latest statistics of the Palestinian Central Bureau of Statistics were used. To achieve the sample size to be included in this study, it was decided a priori that 500 nurses would be invited to participate in this study. The decision to invite this number of nurses for this study was informed by previous studies conducted among nurses and other healthcare providers [39–41].

The study objectives and methodology were explained to the nurses. The nurses had to provide their written informed consent before they could participate in this study. The inclusion criteria were: 1) a practicing nurse, 2) providing written informed consent to participate in this study, and 3) expressing willingness to respond to a questionnaire. Nurses who did not provide a written consent to participate in this study, and those who expressed unwillingness to respond to a questionnaire were excluded from the current study. Participation in this study was voluntary and the nurses were not offered any financial incentives as a compensation for their participation in the current study.

### Assessment tool and validity

The study tool was adopted from previous studies that were conducted among healthcare providers [27, 42–44]. The paper-based questionnaire was handed to potential participants through a personal contact in the healthcare centers/hospitals. The nurses were instructed that the principal investigator and the personal contacts were available and could be contacted in case the nurses needed to clarify any point. In the first section of the questionnaire used in this study, nurses were asked to report their gender. Previous studies reported differences in knowledge, attitudes, and use of protective measures against COVID-19 among male and female healthcare providers [22, 27, 29, 41, 43, 45–47]. The nurses were also asked to self-rate their satisfaction with their financial status, social status, academic achievements, and perceived knowledge about COVID-19 using a Likert-scale of 1–3 (1 = low, 3 = high). Previous studies have shown that satisfaction with financial status, social status, and academic achievements were associated with knowledge, attitudes, and behaviors [48]. During the ongoing pandemic, many healthcare centers suffered shortages of personal protective equipment and healthcare providers had to purchase their own sanitizers, gloves, masks, and other personal protective equipment [49]. Probably, dissatisfaction with one’s financial status might influence purchasing sanitizers, gloves, masks, and other personal protective equipment. Lately, social networks have emerged as an important source of information to the general public as well as to healthcare providers. During the pandemic, the latest news and information about COVID-19 went viral on social media networks [50]. Probably, socially active nurses had larger social networks and could have received more information that influenced their knowledge, attitudes, and use of protective measures against COVID-19. Satisfaction with academic achievements and perceived knowledge about COVID-19 were collected to investigate if there was an association between these variables and performance of the nurses in knowledge, attitudes, and use of protective measures against COVID-19 items. The nurses were also asked to report whether they have contracted COVID-19 before or not. This variable was collected to investigate if contracting COVID-19 affected nurses’ knowledge, attitudes, and use of protective measures against COVID-19. Additionally, nurses were asked to provide their sources of information about COVID-19. In the second section, nurses were asked to respond to a knowledge test of 8 items on the causative agent of COVID-19, signs and symptoms, similarity with flu/cold, treatment, high risk patients, risk for infection, protection, and myths. On each item, the participants had to respond either by true, false, or I do not know. Attitude of nurses was measured using 2 items relevant to finally controlling COVID-19 and confidence in the health authorities in the oPt to win the battle against the disease. On each item, the participants had to choose either disagree, neutral/not sure, or agree. Use of protective measures was examined using 3 items relevant to using soap/sanitizer, physical distancing, and wearing personal protective equipment. On each item, the participants had to report their use either by yes which meant always or most of the time or no which meant not always/most of the time.

The study tool was assessed for face validity by 5 panelists. The panelists who were academician and practicing nurses who had Doctor of Philosophy (PhD) degrees in nursing (*n = 3*), an epidemiologist, and an infectious diseases specialist were asked to rate each item for suitability on a 5-point Likert-scale (1 = not suitable at all, 5 = highly suitable). All items used in the instrument was rated as either suitable or highly suitable by all panelists.

The questionnaire was pilot tested for readability and comprehension with 12 nurses who did not participate in the full study. In this pilot testing, the nurses read the questionnaire and provided their feedback on the clarity and comprehensibility of the items. Based on the feedback received from the nurses in this pilot, some items were reworded to enhance readability and comprehensibility. To ensure stability of scores over a short period of time, the test-retest method was used. A total of 25 nurses who did not participate in the larger study were asked to respond the questionnaire. After a period of 30 min to 1 h, the same 25 nurses were asked to respond to the questionnaire again. Pearson’s correlations were used to correlate the scores of the 25 nurses in the 2 rounds. It was decided *a priori* that a correlation coefficient of more than 80% would be needed to ensure stability of scores over the short time period that was let between the 2 rounds [51–53]. The Pearson’s correlation coefficient was 0.96 (95% CI of 0.91 to 0.98) with a *p* value of < 0.001 which indicated excellent stability of scores. The internal consistency of the items in the questionnaire was assessed using Cronbach’s alpha. Internally consistent tools have a Cronbach’s alpha of ≥0.70 [54]. When all items were included, the Cronbach’s alpha was 0.75. When the Cronbach’s alpha was computed for each domain separately, knowledge items had a Cronbach’s alpha of 0.76, attitude items had a Cronbach’s alpha of 0.97, and use of protective measures had a Cronbach’s alpha of 0.87. Which indicated that the items were internally consistent across all domains.

The items used in the knowledge test were psychometrically evaluated by their difficulty index as calculated by the ratios of correct answers for each item [53, 55, 56]. Questions were psychometrically attributed as: 1) 0% ≤ “very difficult” < 30%, 2) 30% ≤ “difficult” < 60%, 3) 60% ≤ “moderate” < 80%, 4) 80% ≤ “easy” < 90%, and 5) 90% ≤ “very easy” < 100% [53].

### Data analysis

For each knowledge item, the nurses were awarded 1 point for each correct answer and 0 for each incorrect/I don’t know answer. Points were summed (possible points could range from 0 to 8) and were transformed into percentages of correct answers (possible percentages could range from 0 to 100%). Nurses could rate each attitude item on a Likert scale of 1–3. The possible raw ratings (scores) on the attitude items could range from 2 to 6. Attitude scores were transformed into percentages. The practice items were dichotomous (no/yes) and scores could range from 0 to 100%. Data obtained in this study were entered and analyzed statistically using IBM SPSS for Windows, version 21.0 (IBM Inc., Armonk, NY). As the sample size was more than 300, normality of distribution was assessed using absolute skewness and kurtosis values [57, 58]. To fulfil the criteria for normal distribution, the absolute skewness had to fall within the range of − 2.0 and + 2.0 and the absolute kurtosis had to fall within the range − 7.0 and + 7.0. As the criteria for normal distribution were fulfilled, the data were expressed as mean (SD). Differences between knowledge, attitude, and use of protective measures scores among the nurses were investigated using either Student’s t-test or Analysis of Variance (ANOVA) with Bonferroni test as appropriate. Knowledge, attitude, and use of protective measures scores were correlated using Pearson’s correlations. To control potentially confounding variables, predictors of higher knowledge, attitude, and use of protective measures were identified using multiple linear regression analyses. The variables with a *p*-value of < 0.25 in the student’s t-test, ANOVA, and/or Pearson’s correlations were retained in the multiple linear regression models. Enter method was used. For each multiple regression model, the adjusted R-squared with a p-value of < 0.05 was used to evaluate the goodness-of-fit. Tolerance and variance inflation factor (VIF) values were used to assess the multicollinearity of the regression models. Absence of multicollinearity was ensured by tolerance values of > 0.1 and VIF values of close to 1 [59, 60]. In this study, *p*-values of ≤0.05 were considered statistically significant.

### Ethics approval and consent to participate

This study was conducted in adherence with the principles of the Declaration of Helsinki and the ethical principles followed at An-Najah National University. The current study received ethical approval from the Institutional Review Board (IRB) of An-Najah National University. The nurses provided written informed consent before they participated in the current study.

## Results

### Participants’ characteristics

In this, of the 500 nurses invited, 455 (91.0%) completed the questionnaire. The sociodemographic and other variables of the study participants are shown in Table 1. Of the study participants, 285 (62.6%) were female in gender, 385 (84.6%) self-rated their financial status as moderate or high, 430 (94.5%) self-rated their social status as moderately or highly satisfactory, 355 (78%) self-rated their academic achievements as moderately or highly satisfactory, and 425 (93.4%) self-rated their knowledge about COVID-19 as moderately or highly satisfactory. Of the participants, 40 (8.8%) reported that they have contracted COVID-19.

**Table 1:** Participants’ characteristics (*n = 455*)

Characteristic	n	%
**Gender**		
Male	170	37.4
Female	285	62.6
**Self-rated financial status (reported on a Likert-scale of 1–3)**		
Low	70	15.4
Moderate	370	81.3
High	15	3.3
**Self-rated social status (reported on a Likert-scale of 1–3)**		
Low	25	5.5
Moderate	315	69.2
High	115	25.3
**Self-rated academic achievements (reported on a Likert-scale of 1–3)**		
Low	100	22.0
Moderate	310	68.1
High	45	9.9
**Self-rated knowledge about COVID-19 (reported on a Likert-scale of 1–3)**		
Low	30	6.6
Moderate	350	76.9
High	75	16.5
**Have been infected with COVID-19**		
No	415	91.2
Yes	40	8.8

### Sources of knowledge about COVID-19

When the study participate were asked to provide their sources of knowledge about COVID-19, 415 (91.2%) of the participants indicated that they obtained information about COVID-19 through the internet/social media, 230 (50.5%) obtained information about COVID-19 through TV/radio. Friends/family/acquaintances, awareness brochure/leaflet, courses taught at the university, and newspapers/magazines were also cited as sources of information for the participants. Details of the sources of information about COVID-19 are provided in Table 2.

**Table 2:** Sources of information on COVID-19

Source of information	n^a^	%^a^
Through the internet/social media	415	91.2
Through the TV/radio	230	50.5
Friends/family/acquaintances	205	45.1
Awareness brochure/leaflet	195	42.9
During a course taught at the university	90	19.8
From a newspaper/magazine	85	18.7

^a^The participants were able to provide multiple sources; therefore, the number of respondents does not sum to the total number of the study participants and the percentages do not sum to 100%

### Knowledge about COVID-19

The mean knowledge score was 75.7% (SD: 12.4%). Of the nurses, 140 (30.8%) scored 80% and above in the knowledge test. Of the 8 knowledge items, 4 (50.0%) were attributed as “very easy”, 1 (12.5%) was attributed as “easy”, 1 (12.5%) was attributed as “moderate”, and 2 (25.0%) were attributed as “difficult”. No item was attributed as “very difficult”. Of the participants, 390 (85.7%) could correctly identify the causative agent of COVID-19 disease as a virus, 445 (97.8%) could correctly identify the signs and symptoms of COVID-19 disease. However, only 100 (22.0%) of the participants knew that signs and symptoms of COVID-19 disease were similar to those of flu or cold, and 355 (78.0%) knew that there was no current effective treatment for COVID-19 disease. Of the participants, 410 (90.1%) could correctly identify immunocompromised and older individuals as have higher risk for infection and complications of COVID-19, 450 (98.9%) could identify crowded places as source of infection, and 415 (91.2%) knew that using masks appropriately can prevent the spread of COVID-19. However, only 155 (34.1%) knew that antibiotics cannot prevent transmission of COVID-19. Detailed answers of the participants are shown in Table 3.

**Table 3:** Answers of the participants on the 8-item knowledge test

			Answers						
			I don’t know		True		False		
#	Item	Correct answer	n	%	n	%	n	%	Difficulty index
1	COVID-19 is a viral infection	True	55	12.1	**390**	**85.7**	10	2.2	Easy
2	The possible signs and symptoms of COVID-19 are fever, sore throat, cough, myalgia and shortness of breath	True	5	1.1	**445**	**97.8**	5	1.1	Very easy
3	Signs and symptoms of COVID-19 can be similar as flu or cold	True	60	13.2	**100**	**22.0**	295	64.8	Difficult
4	Currently, there is no effective treatment for COVID-19, but early symptomatic and supportive treatment can help most patients recover from the infection	True	70	15.4	**355**	**78.0**	30	6.6	Moderate
5	People with a compromised immune system and old age people are at more risk of developing the infection	True	10	2.2	**410**	**90.1**	35	7.7	Very easy
6	People in crowded places are at increased risk of getting affected by the disease	True	5	1.1	**450**	**98.9**	0	0.0	Very easy
7	If appropriately used, medical masks can prevent the spread of infection	True	5	1.1	**415**	**91.2**	35	7.7	Very easy
8	Taking antibiotics can prevent the transmission of COVID-19	False	190	41.8	110	24.2	**155**	**34.1**	Difficult

Correct answers are in boldface

### Attitude of the participants with regard to COVID-19

The mean attitude score was 75.1% (SD: 17.7%). Of the nurses, 215 (47.3%) scored 80% and above on the attitude items. Of the participants, 290 (63.7%) were positive that COVID-19 will finally be successfully controlled and only 115 (25.3%) had confidence that the health authorities in the oPt would win the battle against COVID-19. Detailed responses of the participants are shown in Table 4.

**Table 4:** Attitude of the participants with regard to COVID-19

		Disagree		Neutral/Not sure		Agree	
#	Item	n	%	n	%	n	%
1	Do you agree that COVID-19 will finally be successfully controlled?	20	4.4	145	31.9	290	63.7
2	Do you have confidence that the health authorities in the oPt can win the battle against COVID-19?	155	34.1	185	40.7	115	25.3

*oPt* Occupied Palestinian territory

### Use of protective measures against COVID-19

The mean use of protective measures against COVID-19 score was 91.6% (SD: 18.2%). Of the nurses, 430 (94.5%) scored 80% and above on the practice items. Of the participants, 415 (91.2%) reported using soap or sanitizer to wash their hands and faces, 395 (86.8%) avoided unnecessary close contact and practiced physical distancing, and 440 (96.7%) reported wearing necessary personal protective equipment during interaction with the patients. Detailed responses of the participants are provided in Table 5.

**Table 5:** Use of protective measures against COVID-19

		No^**a**^		Yes^**b**^	
#	Item	n	%	n	%
1	I am using soap or sanitizer to wash hands and face	40	8.8	415	91.2
2	I avoid unnecessary close contact and practice physical distancing and keep at least 1-m distance from patients and other healthcare workers	60	13.2	395	86.8
3	During interaction with the patient (including COVID-19 patient), I wear the necessary personal protective equipment such as masks, gloves, and gown, etc	15	3.3	440	96.7

^a^No: Not always/most of the time

^b^Yes: Always/most of the time

### Correlation between knowledge, attitude, and practice scores

Spearman’s correlations showed that there was a significant low positive correlation between knowledge scores and use of protective measures against COVID-19 (Pearson’s r = 0.27, *p* value < 0.001). Similarly, there was a significant low positive correlation between attitude scores and use of protective measures against COVID-19 (Pearson’s r = 0.13, *p*-value = 0.007). Details of the correlations between knowledge, attitude, and use of protective measures against COVID-19 are shown in Table 6.

**Table 6:** Correlations between knowledge, attitude, and practice scores

Score	Knowledge		Attitude		Use of protective measures	
	rho	***p***-value	rho	***p***-value	rho	***p***-value
**Knowledge**	–		0.08	0.095	0.27	< 0.001
**Attitude**	0.08	0.095	–		0.13	0.007
**Use of protective measures**	0.27	< 0.001	0.13	0.007	–	

### Differences in knowledge, attitude, and use of protective measures against COVID-19 among the nurses

Table 7 shows differences in knowledge, attitude, and use of protective measures against COVID-19 among the participants. In this study, knowledge scores were significantly lower for nurses who were male, self-rated their financial status as low, self-rated their social life as low, and self-rated their knowledge about COVID-19 as low compared to nurses who were female and those who self-rated their financial status, social life, and knowledge about COVID-19 differently (Table 7). Attitude scores were significantly lower for nurses who were female, having contracted COVID-19 before, self-rated their financial status as high, self-rated their academic achievements as low, and self-rated their knowledge about COVID-19 as high, compared to nurses who were male, have not contracted COVID-19, self-rated financial status, academic achievements, and knowledge about COVID-19 differently (Table 7). Use of protective measures scores were significantly lower for nurses who self-rated their academic achievements as low and self-rated their knowledge about COVID-19 as low compared to those who self-rated their academic achievements and knowledge about COVID-19 differently (Table 7).

**Table 7:** Differences in knowledge, attitude, and use of protective measures against COVID-19 among the nurses

			Knowledge					Attitude					Use of protective measures				
Variable	n	%	Mean	SD	***p***-value	Pearson’s r	***p***-value	Mean	SD	***p***-value	Pearson’s r	***p***-value	Mean	SD	***p***-value	Pearson’s r	***p***-value
**Gender**																	
Male	170	37.4	73.2	10.1	0.001	0.16	0.001	79.4	16.2	< 0.001	−0.19	< 0.001	90.2	20.7	0.213	0.06	0.213
Female	285	62.6	77.2	13.3				72.5	18.1				92.4	16.6			
**Self-rated financial status (reported on a Likert-scale of 1–3)**																	
Low	70	15.4	70.5	7.7	< 0.001	0.12	0.008	73.8	16.4	< 0.001	−0.06	0.195	90.5	19.8	0.176	0.06	0.208
Middle	370	81.3	76.9	13.0				76.1	17.8				91.4	18.2			
High	15	3.3	70.8	6.1				55.6	8.1				89.9	18.3			
**Self-rated social life (reported on a Likert-scale of 1–3)**																	
Low	25	5.5	67.5	10.2	0.002	0.13	0.006	70.0	16.7	0.195	0.00	0.967	86.7	27.2	0.149	−0.02	0.710
Middle	315	69.2	74.3	13.2				75.9	17.0				92.6	17.3			
High	115	25.3	77.2	9.5				73.9	19.6				89.9	18.3			
**Self-rated academic achievements (reported on a Likert-scale of 1–3)**																	
Low	100	22	74.4	9.3	0.403	0.03	0.491	70.0	18.0	0.004	0.14	0.002	86.7	22.2	0.009	0.12	0.012
Middle	310	68.1	76.2	12.9				76.3	16.6				93.0	16.0			
High	45	9.9	75.0	14.6				77.8	22.5				92.6	21.2			
**Self-rated knowledge about COVID-19 (reported on a Likert-scale of 1–3)**																	
Low	30	6.6	70.8	14.1	0.002	−0.04	0.452	75.0	8.5	0.023	−0.10	0.033	77.8	25.3	0.000	0.05	0.246
Middle	350	76.9	76.8	12.0				76.2	18.4				93.3	16.5			
High	75	16.5	72.5	12.3				70.0	16.4				88.9	20.0			
**Have been infected with COVID-19**																	
No	415	91.2	75.5	12.8	0.192	0.06	0.192	76.1	17.8	< 0.001	−0.18	< 0.001	91.2	18.7	0.122	0.07	0.122
Yes	40	8.8	78.1	5.5				64.6	13.2				95.8	11.2			

### Factors predicting higher knowledge, attitude, and use of protective measures

Multiple linear regression showed that higher knowledge scores were predicted by being female (*p*-value = 0.004) and self-rating social status as high (*p*-value = 0.005). Attitude scores were predicted by being female (*p*-value = 0.005), self-rating academic achievement as high (*p*-value = 0.007), and having contracted COVID-19 (*p*-value = 0.001) (Table 8). Other variables were no longer significantly associated. Use of protective measures scores were predicted by self-rating academic achievements as high (*p*-value = 0.010) (Table 8). Other variables were no longer significantly associated. The tolerance values of the regression models were > 0.1 (in the range of 0.88 to 0.99) and the VIF values were in close to 1 (in the range of 1.01 to 1.14). These values indicated absence of multicollinearity among the predictors.

**Table 8:** Multiple linear regression analyses of association between variables of the participants with knowledge, attitude, and use of protective measures against COVID-19

						Collinearity	
Variable	Unstandardized Coefficients	SE	Standardized Coefficients	t	***p***-value	Tolerance	VIF
**Knowledge**							
Gender	3.62	1.25	0.14	2.90	0.004	0.88	1.14
Self-rated financial status	2.19	1.43	0.07	1.53	0.126	0.91	1.10
Self-rated social life	3.12	1.10	0.13	2.84	0.005	0.99	1.01
Self-rated academic achievements	1.29	1.04	0.06	1.24	0.216	0.97	1.03
Self-rated knowledge about COVID-19	−1.21	1.23	−0.05	− 0.99	0.324	0.96	1.04
Have been infected with COVID-19	2.32	2.06	0.05	1.13	0.261	0.94	1.06
Constant	56.43	5.31		10.62			
**Attitude**							
Gender	−4.96	1.77	− 0.14	−2.80	0.005	0.88	1.14
Self-rated financial status	−1.38	2.02	−0.03	− 0.68	0.496	0.91	1.10
Self-rated social life	−0.06	1.56	0.00	−0.04	0.969	0.99	1.01
Self-rated academic achievements	4.02	1.48	0.13	2.73	0.007	0.97	1.03
Self-rated knowledge about COVID-19	−2.48	1.74	−0.07	−1.43	0.155	0.96	1.04
Have been infected with COVID-19	−9.41	2.92	−0.15	−3.22	0.001	0.94	1.06
Constant	93.77	7.52		12.47			
**Use of protective measures**							
Gender	1.89	1.87	0.05	1.01	0.313	0.88	1.14
Self-rated financial status	1.95	2.14	0.05	0.91	0.362	0.91	1.10
Self-rated social life	−0.25	1.65	−0.01	− 0.15	0.881	0.99	1.01
Self-rated academic achievements	4.03	1.56	0.12	2.58	0.010	0.97	1.03
Self-rated knowledge about COVID-19	1.64	1.84	0.04	0.89	0.373	0.96	1.04
Have been infected with COVID-19	3.66	3.09	0.06	1.19	0.236	0.94	1.06
Constant	70.37	7.96		8.84			

*SE* Standard error, *t* t statistic, *VIF* Variance inflation factor. Male, low financial status, low social life, low academic achievements, low knowledge about COVID-19, and not having contracted COVID-19 were the reference categories for gender, self-rated financial status, self-rated social life, self-rated academic achievements, self-rated knowledge about COVID-19, and have been infected with covid-19, respectively

## Discussion

In the present multicenter study, knowledge, attitude, and use of protective measures against COVID-19 among nurses practicing in healthcare centers/hospitals across the West Bank of the oPt during the ongoing pandemic were assessed. The study highlighted some high awareness areas, moderately optimistic attitude, and some adequate use of protective measures against COVID-19 among nurses. Additionally, predictors of high knowledge, positive attitude, and appropriate use of protective measures against COVID-19 were also identified. This is the first study among nurses with regard to COVID-19. The results of this study might shed light on the current behavior of nurses during the ongoing pandemic. Findings of this study are informative to decision makers in healthcare authorities and professional groups for designing measures and appropriate interventions to increase knowledge, positive attitude, and promote adequate use of protective measures against COVID-19 that might protect nurses from contracting COVID-19 during the ongoing global pandemic and other future viral pandemics.

Although it is difficult to define adequate knowledge about COVID-19, less than a third (30.8%) of the nurses scored 80% and above in the knowledge test. Findings of this study might indicate that knowledge about COVID-19 among the majority of the nurses was less than optimal. In this study, the nurses obtained their information on COVID-19 from different sources, notably, the internet/social media and TV/radio. Previous studies reported that healthcare providers including nurses were high users of different social media networks [61]. Healthcare providers including nurses often subscribe to official pages of professional health organizations/societies that could be news outlets for many nurses. Additionally, during the lockdown and “stay at home” orders, people including healthcare providers followed the latest news about the pandemic. Nurses are also increasingly using social media networks to communicate with their peers. Latest information with regard to COVID-19 often go viral on social media networks. Although the power of the internet in spreading knowledge was recognized long time ago, social media networks and different online learning platforms were extensively used during the pandemic by almost all educational institutions during the pandemic [62]. Decision makers might use these platforms or other suitable educational channels to increase knowledge of nurses with regard to COVID-19.

In this study, knowledge scores of female nurses were higher than those of male nurses. This probably meant that female nurses were more knowledgeable about COVID-19 compared to their male counterparts. In a study using similar knowledge items among pharmacists in Pakistan, female pharmacists reported higher knowledge scores compared to their male counterparts [27]. These findings were consistent with those reported in previous studies in which there were differences in knowledge between male and female nurses in different countries including knowledge about COVID-19 [22, 29, 41, 43, 45–47]. Traditionally, nursing was viewed as more suitable for female nurses, however, recent qualitative studies have reported that male nurses perceive the profession equally suitable compared to their female counterparts [63]. Probably, more studies are needed to understand why female nurses tend to score more in knowledge tests compared to male nurses and how to improve knowledge of male nurses in certain domains to thrive in their professional development. In this study, nurses who self-rated their social life as high had higher scores than nurses who did not self-rated their social life as high. Probably, socially active nurses have larger networks of acquaintances to interact with and exchange information about COVID-19. Such interactions might have expanded their knowledge of COVID-19 through knowledge seeking behavior and exposure to information about COVID-19 [64–67]. Findings of this study were consistent with those reported among healthcare providers [27, 29].

Less than half (47.3%) of the nurses who participated in this study scored 80% and above on the attitude items. Healthcare providers in Pakistan, China, and Jordan generally expressed positive attitudes with regard to containing the pandemic [27, 42, 43, 47, 68, 69]. Nurses who contacted COVID-19 were less positive in this regard compared to the nurses who did not contract the disease. In this study, severity of the symptoms experienced by those who contracted the disease was not assessed. This precluded investigating whether severe symptoms might have affected attitudes of the nurses or not. More than half of the nurses (63.7%) agreed that COVID-19 will finally be successfully controlled. When the nurses were asked about their confidence that health authorities in the oPt could win the battle against COVID-1, the majority of the nurses (74.8%) either disagreed or were neutral/not sure. In this study, the nurses seemed to have more faith in the global efforts to contain COVID-19 compared to the efforts of the health authorities in the oPt. Nurses who self-rated their academic achievements as high expressed more positive attitude compared to nurses who did not self-rate their academic achievements as high. During their academic program, nurses are offered courses in pathology, pharmacology, microbiology/immunology/virology, and public health. Additionally, nurses receive higher volumes of hospital-based training as they progress into later stages of their nursing program. Therefore, nurses are expected to gain more knowledge relevant to diseases, viruses, signs and symptoms of infections, treatments, disease related risk factors, and infection control techniques [70]. This might help developing positive attitude toward science-based containment efforts.

Use of protective measures against COVID-19 positively correlated with knowledge and attitude scores. Taken together, these results might at least in part indicate that good knowledge supported by positive attitude might promote adequate use of protective measures against COVID-19 among nurses. Probably, appropriately designed educational interventions might be helpful in improving awareness of nurses on COVID-19 and similar viruses, increasing positive attitude toward containment approaches, and promoting adequate use of protective measures against COVID-19. Additionally, improving financial and social life conditions of nurses could also improve knowledge, attitude, and use of adequate protective measures against COVID-19 and similar viruses.

### Strengths and limitations of the study

This is the first study among nurses in general and among healthcare providers in the oPt with regard to their knowledge, attitude, and use of protective measures against COVID-19 during the ongoing pandemic. In this study, the response rate was 91.0%. The response rate obtained in this study was high when compared to response rates reported in previous studies in which a questionnaire was used as a study tool among healthcare providers including nurses [39, 41, 48, 52]. Interestingly, the number of nurses who responded to the questionnaire was larger than the sample size needed for this study. This should have minimized the potential bias associated with low response rates. Additionally, the nurses who responded in this study were from both genders and had variable financial, social life, academic achievements, and self-rated knowledge of COVID-19. The sample also included nurses who previously contracted COVID-19. This diversity might have added validity, depth, and width to the findings of this study. Although the tool used in this study was adopted from previous studies, the tool was revalidated in a pilot testing using appropriate tests [27, 42–44]. Findings of the pilot testing phase indicated that the tool was suitable to be used to assess knowledge, attitude, and use of protective measures against COVID-19 nurses [51–53]. This might have allowed exposing the current knowledge, attitude, and use of protective measures against COVID-19 among nurses practicing across the healthcare centers/hospitals in the West Bank of the oPt.

The findings of this study should also be interpreted considering the following limitations. First, this study was a cross-sectional study. The findings might change with time and knowledge might increase as the pandemic continue unfolding. Additionally, the findings could have been more interesting should an intervention to improve knowledge, attitude, and use of protective measures was attempted. However, findings of this study might be informative to decision makers who wish to intervene by designing appropriate measures aiming to protect future nurses by improving knowledge, correcting attitude, and promoting adequate use of protective measures against COVID-19 among nurses. Second, the self-rated financial status, self-rated social status, self-rated academic achievements, and self-rated knowledge about COVID-19 were measured using a three-point Likert scale. Although the Likert scale is popularly used in medical research, the number of scale points to be used is still highly controversial [71]. Previous studies have used Likert scales with a number of points that ranged from 3 to 11. In a previous study, Leung administered the Rosenberg Self-Esteem Scale among 1217 students in Macau using different number of points and showed that there were no significant differences in Cronbach’s alpha, item–item correlations, item–total correlations, factor loadings, mean scores, and standard deviation of the scores [71]. Although the study of Leung advocated the use of large number of points (> 6 points), five-point Likert scales are commonly used in medical research. In this study, the use of a three-point Likert scale might have influenced the number of nurses who self-rated their financial status, social status, academic achievements, and knowledge about COVID-19 as moderate. This could have limited generalization and/or comparison of the findings to other settings. Third, the number of items measuring knowledge, attitude, and use of protective measures against COVID-19 among nurses with regard to COVID-19 was relatively small. Additionally, attitudes of the nurses were measured using only 2 items. However, the tool was previously used to assess knowledge, attitudes, and practice among healthcare providers in other settings studies [27, 42–44]. Despite the inherent disadvantages, the use of small number of items in a questionnaire has many advantages including increasing participation, avoiding participant fatigue, and saving the time of the participants [72, 73]. Additionally, the items used to assess knowledge ranged from very easy to difficult. However, no question was attributed as very difficult in this study. Fifth, the use of protective measures against COVID-19 items collected perceived practice behavior. Although, social desirability bias cannot be excluded, it is noteworthy mentioning that the study participants were nurses who cared for infected patients during an ongoing pandemic. This could also, at least in part, explain the reportedly high use of use of protective measures against COVID-19. Finally, a nonprobability sampling technique was used to recruit the nurses to this study. Compared to probability sampling, nonprobability sampling techniques are inherently biased. This might limit generalization of the findings to the entire population of nurses. Fourth, knowledge of the nurses might have been underestimated as a results of recall bias. During the pandemic, nurses as well as other healthcare providers were exhausted and had to work for extended shifts. Previous studies conducted elsewhere including neighboring Jordan have reported high prevalence of burnout among healthcare providers [12–14, 74]. It is possible that the exhaustive work conditions during the pandemic have affected the results.

## Conclusion

Findings of this study suggested that nurses in the oPt had adequate knowledge, relatively optimistic attitude, and appropriately used protective measures against COVID-19 during the ongoing pandemic. Knowledge, attitude, and use of protective measures against COVID-19 among nurses should continuously be updated as information unfold during the ongoing pandemic. More efforts are still needed to ensure protection of healthcare providers including nurses from contracting COVID-19.

## Supplementary Information

**Additional file 1: Supplementary Table S1.** Adherence to the guidelines of reporting of cross-sectional studies in which a questionnaire was used as the study tool [1–3].

## Data Availability

All data relevant to this study are included within the manuscript or provided as supplementary materials.

## Abbreviations

2019-nCoV: Novel coronavirus 2019
CI: Confidence interval
COVID-19: Coronavirus disease 2019
IQR: Interquartile range
IRB: Institutional Review Board
MERS: Middle East respiratory syndrome
R_0_: Reproductive number
SARS: Severe acute respiratory syndrome
SARS-CoV-2: Severe acute respiratory syndrome coronavirus 2
VIF: Variance inflation factor
WHO: World Health Organization
