# Supplementary material for: Knowledge, attitude, and use of protective measures against COVID-19 among nurses: a questionnaire-based multicenter cross-sectional study
Source: BMC Nurs. 2021 Sep 7;20:163. doi: 10.1186/s12912-021-00689-x (PMC8422377; doi:10.1186/s12912-021-00689-x)
Supplement: Supplementary file 1 — Additional file 1: Supplementary Table S1. Adherence to the guidelines of reporting of cross-sectional studies in which a questionnaire was used as the study tool [1–3]. [file 12912_2021_689_MOESM1_ESM.docx]

**Supplementary materials for the manuscript:**

**Knowledge, attitude, and use of protective measures against COVID-19 among nurses: A questionnaire-based multicenter cross-sectional study**

Ramzi Shawahna^1,2*^

^1^Department of Physiology, Pharmacology and Toxicology, Faculty of Medicine and Health Sciences, An-Najah National University, Nablus, Palestine

^2^An-Najah BioSciences Unit, Centre for Poisons Control, Chemical and Biological Analyses, An-Najah National University, Nablus, Palestine

**^*^Correspondence:**

Ramzi Shawahna, PhD, Department of Physiology, Pharmacology and Toxicology, Faculty of Medicine & Health Sciences, New Campus, Building: 19, Office: 1340, An-Najah National University, P.O. Box 7, Nablus, Palestine

Phone: + (970) 923 45113 ext 2772

Phone: + (970) 92349739

Email: [ramzi_shawahna@hotmail.com](mailto:ramzi_shawahna@hotmail.com)

**Supplementary Table S1:** Adherence to the guidelines of reporting of cross-sectional studies in which a questionnaire was used as the study tool [[1-3](#_ENREF_1)]

| **Checklist Item** | **Explanation** | **Place in the manuscript** |
| --- | --- | --- |
| **Title and abstract** |  |  |
| Design of the study stated | Are the words 'questionnaire' or 'survey' stated in the title and/or abstract? | The term “questionnaire” was mentioned in the title and the abstract as well as in other sections of the manuscript |
| **Introduction/Background** |  |  |
| Background provided | Did the authors present a well-written background to their research? | A background to the study was provided under Background section in this manuscript |
| Purpose/aim of paper explicitly stated | Did the authors identify a specific purpose, aim, goal, or objective of the study? | The objectives of the study were provided in the last paragraph of the Background section |
| **Tool of measurement** |  |  |
| Description of the questionnaire | Did they provide access to the questionnaire items used in the study in either the article, appendices, or an online supplement? | Items in the questionnaire were described. References to the questionnaire were provided. |
| References to original work provided | Did they provide a reference to the existing tool (questionnaire) that they used? | References to the previous works in which the questionnaire was used were provided under (Assessment tool and validity) |
| Psychometric properties | Psychometric properties = (were the reliability and psychometric of the existing questionnaire mentioned?) | Reliability and psychometric were provided under (Assessment tool and validity) |
| Description of the scoring procedures provided | Did papers which used survey instruments that required scoring provide a description of the scoring procedures? | Provided under (Data analysis) |
| **Recruitment process and sample description** |  |  |
| Description of survey population and sample frame | Survey population = the main target of the study. Sample frame = the methods they used to reach this target. For instance, if a study wanted to detect the prevalence of hypertension in elderly people above 65 years old, and researchers went to 10 elderly care centers and randomly recruited 50 old men from the registry of each of those centers. This means that our survey population is elderly people above 65, and the sample frame is the 10 care centers registry. | Provided under (Study participants and sampling) |
| Description of representativeness of the sample | Is a description of whether the sample will represent the whole population provided? | Provided under (Study participants and sampling) |
| Sample size calculation or rationale/justification presented | Did they mention a description of their sample size calculation, such as providing a formula or a rationale? | Provided under (Study participants and sampling) |
| Incentives | Were any incentives offered (e.g., monetary, prizes, or non-monetary incentives such as an offer to provide the survey results)? | Provided under (Study participants and sampling) |
| **Survey Administration** |  |  |
| Mandatory/voluntary | Was it a mandatory survey to be filled in by every visitor who wanted to enter the Website, or was it a voluntary survey? | Provided under (Study participants and sampling) |
| Dates | In what timeframe were the data collected? | Provided under (Study design and the questionnaire) |
| **Analysis** |  |  |
| Methods of data analysis | Was a description of the variables that were analyzed, how they were manipulated, and the statistical methods that were used provided? | Provided under (Data analysis) |
| **Results** |  |  |
| Response rate reported | Was response rate reported? | Provided under (Participants’ characteristics) |
| Results clearly presented | _ | Provided in the Results section |
| Results address objectives | _ | Provided in the Results section |
| **Discussion** |  |  |
| Results summarized referencing study objectives | _ | Provided in the first paragraph of the Discussion section |
| Strengths of the study stated | _ | Provided under Strength and Limitations |
| Limitations of the study stated | _ | Provided under Strength and Limitations |
| Generalizability of results discussed | Did they include any discussion on the generalizability of their results? | Discussed under Strength and Limitations |
| **Ethical Quality Indicators** |  |  |
| Study funding reported |  | Provided in the Declarations |
| Research Ethics Board (REB) review reported |  | Provided in the Declarations |
| Subject consent procedures reported |  | Provided in the Declarations |

**References**

1. Turk T, Elhady MT, Rashed S, Abdelkhalek M, Nasef SA, Khallaf AM, Mohammed AT, Attia AW, Adhikari P, Amin MA *et al*: **Quality of reporting web-based and non-web-based survey studies: What authors, reviewers and consumers should consider**. *PLOS ONE* 2018, **13**(6):e0194239.

2. Sharma A, Minh Duc NT, Luu Lam Thang T, Nam NH, Ng SJ, Abbas KS, Huy NT, Marusic A, Paul CL, Kwok J *et al*: **A Consensus-Based Checklist for Reporting of Survey Studies (CROSS)**. *Journal of general internal medicine* 2021.

3. von Elm E, Altman DG, Egger M, Pocock SJ, Gotzsche PC, Vandenbroucke JP, Initiative S: **The Strengthening the Reporting of Observational Studies in Epidemiology (STROBE) statement: guidelines for reporting observational studies**. *PLoS medicine* 2007, **4**(10):e296.
